# Emergent Magnetism as a Cooperative Effect of Interactions and Reservoir

**DOI:** 10.1021/acs.jpclett.3c00526

**Published:** 2023-05-30

**Authors:** M. Shiranzaei, S. Kalhöfer, J. Fransson

**Affiliations:** Department of Physics and Astronomy, Uppsala University, Box 516, 751 20 Uppsala, Sweden

## Abstract

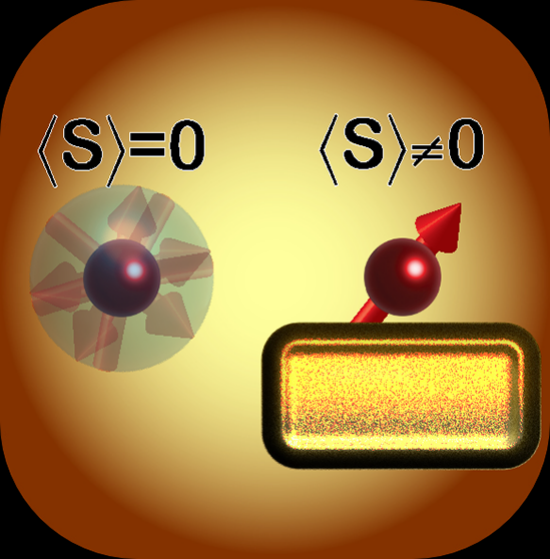

Closed shell molecular structures are under normal conditions time-reversal invariant. Experimental evidence points, however, toward that this invariance may be locally violated when the structure is in contact with a particle reservoir. The mechanisms behind such local symmetry breaking are not clear by any means. By considering a minimal model for a closed shell structure, here we propose that the symmetry breaking may result from a combination of internal and/or external interactions. It is shown that a magnetic moment of a localized electron level can be generated and maintained under the influence of such a combination. The theoretical results should cast new light on the mechanisms that may form magnetic properties in molecular compounds.

Molecules, as well as single atoms, which are in closed shell configurations when isolated, can acquire a magnetic state when, e.g., immersed in solution,^1,2^ attached on a surface,^3−9^ or being in embedded in clusters comprising several components.^10−17^ Such properties can be exploited in, for instance, anomalous Hall devices,^18−20^ electron spin resonance,^21,22^ exploration of superconductivity in the presence of spin impurities^23^ giving rise to Yu-Shiba-Rusinov states,^24,25^ and in structures with properties, such as coercivity^13−17^ and spin-filtering,^16,26−28^ that strengthen with temperature.

The origin of the magnetic state in the closed shell configuration can be effectively summarized as an interplay between the Pauli exclusion principle and the Hund’s rules. Although these rules with some success can be employed also in a more general context, questions about the emergence of magnetic states in molecules that are normally regarded as nonmagnetic inevitably arise. For instance, chiral molecules provide urgent examples of closed shell structures which, nevertheless, display magnetic properties when in contact with otherwise nonmagnetic metals, see, e.g., refs (8, 18, 19, 22, 28, and 29).

In this article we address the issue of the emergence of a magnetic state in or in the proximity of a local electronic structure when it is being exposed to an external environment. We begin by demonstrating that a spin degenerate molecular level may become spin-polarized if two conditions are met. First, there should exist internal molecular interactions which have the potential to break the time-reversal symmetry, and second, the molecular level must be in contact with an external reservoir. We show that the nature of the reservoir, whether it is Fermionic or Bosonic, is secondary. This observation, hence, implies that also molecules in a purely thermal environment may be spontaneously polarized.

As a corollary result of these conditions, we also show that the spin-degeneracy of a localized electron may be broken by a spin-dependent coupling to a purely Bosonic reservoir. Breaking of the spin-degeneracy requires, however, the presence of both spin-conserving *and* spin-nonconserving coupling. In this model we, furthermore, demonstrate the emergence of a nonvanishing magnetic moment and an associated crossover temperature at which this moment undergoes a sign change.

We explain our findings to be a result of confluent interactions since these results cannot be obtained in a system with a single type of interaction. For simplicity, assume that there are two sources of interactions which can be formulated through the quantities *V*_0_σ^0^ and **V**_1_·**σ**, where σ^0^ and **σ** are the 2 × 2-unit matrix and vector of Pauli spin matrices. When these two interactions coexist, the effective interaction changes the spectrum as , which opens up the possibility to break the spin-degeneracy whenever both *V*_0_ and **V**_1_ contribute.

We notice that the nature of the interactions may comprise electron–electron and electron–phonon type of interactions but may also stem from, e.g., light–matter interactions of different kinds. The important feature that has to be recognized in this context is the presence of components with different symmetries with respect to the spin degrees of freedom. In this sense we stipulate that there should be one component that couples to the electron charge and one to the electron spin.

As a philosophical remark, our results are important since they challenge the widespread view that we can interpret measurements in terms of subsystems where the environment has a negligible effect, and we present a concrete example where this is not the case. Despite that we are taught in our scientific training that a measurement inevitably influences the properties of the sample, both interpretations of experimental results as well as theoretical descriptions are many times based on complete negligence of the reservoir to which the sample is connected.

The purpose here is to evaluate the magnetic moment ⟨**m**_0_⟩ of a localized electron represented by the spectrum **ε** = ε_0_σ^0^ + **ε**_1_·**σ**, where ε_0_ and **ε**_1_ denote the energies corresponding to the spin-independent and spin-dependent degrees of freedom. Here, the latter is a three component vector, , in some normalized orthogonal basis , which accounts for, e.g, spin–orbit interactions and local spin-anisotropy. The model corresponding to this spectrum can be written , where  denotes the spinor for the localized state.

In order to enable a general treatment of the local properties, we calculate the expectation value of the magnetic moment ⟨**m**⟩ in terms of the Green function **G**_LS_ for the local electron through the relation , where  denotes the retarded Green function and *f*(ω) is the Fermi–Dirac distribution function, whereas sp is the trace over spin 1/2 space. The equation of motion for **G**_LS_ can be cast in the Dyson-like form
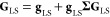
1where **g**_LS_ = **g**_LS_(*z*) = (*z* – **ε**)^−1^, , is the bare Green function defined by , whereas **Σ** denotes the self-energy caused by the interactions the local electron is subject to. In this context, one can notice that the self-energy has (i) an energy dependence, **Σ** = **Σ**(*z*), and (ii) can be written in the form **Σ** = Σ_0_σ^0^ + **Σ**_1_·**σ**, which are natural conditions for spin 1/2 particles. Physically, this partitioning represents the charge- (Σ_0_) and spin-dependent (**Σ**_1_) components of the interactions. However, in addition we shall make the replacement Σ_0_ → *V* + Σ_0_. In this construction, *V* may define a contribution caused by hybridization between the localized state and an external reservoir, whereas the self-energy **Σ** may be attributed to internal or external interactions associated with the localized electron. There is, nevertheless, nothing that prevents the opposite association of *V* and **Σ**, that is, that the former belongs to the molecule and the latter represents the interactions with the environment, as we shall see in the concrete example below.

Summarizing these facts, it is straightforward to write the retarded/advanced Green function as
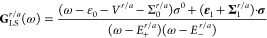
2with the poles
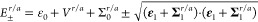
3Of particular interest here is the component comprising the Pauli matrices, since only this term can contribute under the trace . Indeed, using the notation **G**_LS_ = *G*_0_σ^0^ + **G**_1_·**σ**, it can be seen that . Here,
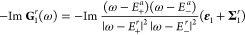
4

In order to sort out the origin of the induced magnetic moment, we set
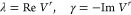
5a
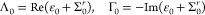
5b
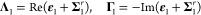
5cand keep in mind that  and . The lesser Green function can, then, be written
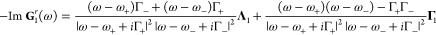
6where ω_±_ = λ + Λ_0_ ± Λ_1_ and Γ_±_ = γ + Γ_0_ ± Γ_1_.

As we wish to determine the origin of the magnetic moment, assume, for the sake of argument, that  strongly peaks at the resonance energies ω_±_, while it is nearly vanishing off resonance. This assumption is justified whenever the broadening Γ_±_ is small in a neighborhood around ω_±_. Then, the magnetic moment can be estimated by approximately
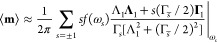
7Assuming, furthermore, that the self-energy strongly peaks at the energy ε_0_ + ω_0_, which does not coincide with either of ω_±_, then, one can notice that Γ_0_(ω_±_) ≈ 0 and **Γ**_1_(ω_±_) ≈ 0, such that Γ_*s*_ → γ. Implementing this observation, it can be seen that the magnetic moment reduces to
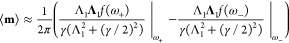
8It should be mentioned that the energy ω_0_ is associated with the energy of the internal interactions captured in **Σ**.

Here, we stress the parameters **Λ**_1_ = **Λ**_1_(ω), **Γ**_1_ = **Γ**_1_(ω), etc. and that they acquire different values at the resonances ω = ω_±_. Hence, in the limit **ε**_1_ = 0, this calculation leading to eq 8 demonstrates that, despite the simplicity inferred, the result comprises a fundamentally important feature of the composite system discussed here. Namely, while the internal interactions, which lead to the self-energy **Σ**, provides an energy dependent shift of the electron resonances and their corresponding lifetimes, as well as an induced finite spin-splitting, and while the coupling between electrons in the localized level and the reservoir contributes to the level broadening of the local resonances, it is only when those two mechanisms are present simultaneously that a finite magnetic moment can be induced and maintained in the localized level.

The implications of this result should have bearing on the interpretation of experimental results, as well as, how a theoretical account for a phenomenon can be made irrelevant by exclusion of effects from the environment. In magnetism, for instance, many types of interactions which, at first sight, may appear unrelated may actually play a nontrivial role for the stabilization of the ordered state.^16,17,30^ The magnetic signatures observed after adsorbing nonmagnetic molecules onto the metallic surface^18,20,25^ stem from mechanisms that are unlikely to be captured within the conventional theory for magnetism.

It is by now established that time-reversal symmetry may be broken by inelastic scattering.^31−33^ Therefore, we consider a simplified example that may be used to illustrate a possible experimental outcome for single molecules in contact with a thermal reservoir. Such a system can be modeled by the Hamiltonian , where  denotes the valence state in the molecule, whereas  represents the thermal reservoir in which  (*b*_**q**_) creates (annihilates) a phonon at the energy ω_**q**_. The electron–phonon coupling is provided through the term
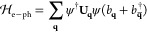
9where the coupling parameter **U**_**q**_ = *u*_0**q**_σ_0_ + **u**_1**q**_·**σ**, whereas . In addition to *u*_0**q**_ which defines a generic coupling between charge and vibrational modes, **u**_1**q**_ denotes a vibrationally induced spin–orbit coupling.^27,34,35^ Here, σ_0_ and **σ** denote the 2 × 2 identity and vector of Pauli matrices, respectively. It should be noticed that the vibrationally induced spin–orbit coupling **u**_1**q**_, in general, is nonvanishing in structures with broken inversion symmetry. Hence, in the present discussion we assume that those requirements are met in the structure.

The processes associated with the terms  and  are illustrated in Figure 1a,b, respectively. In processes of the former kind, the electrons emit or absorb phonons such that the total charge undergoes a transition to the emission or absorption state. By contrast, in processes of the latter, in which both charge and spin are coupled to the phonons, the emission and absorption processes are accompanied by electronic spin-flips and spin-dependent rates.

**Figure 1:**
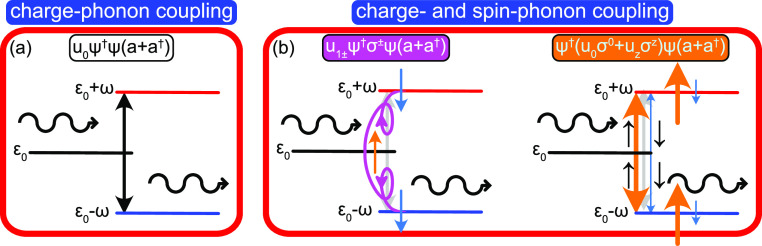
Illustration of the electron–phonon processes involving (a) only charge and (b) both charge and spin. By emission or absorption of phonons, the total charge undergoes transitions to the states at the energies ε_0_ – ω and ε_0_ + ω, respectively. (a) For a coupling solely between the charge and phonons, there is no spin related process. (b) For a coupling that involves both charge and spin, the transitions may be accompanied by spin-flip and spin-dependent rates.

The magnetic moment ⟨**M**_mol_⟩ is related to the retarded single electron Green function , which is given by the Dyson-like equation in eq 1, where the self-energy **Σ** accounts for the phonon assisted absorption and emission processes illustrated in Figure 1. The structure of the model allows us to factorize the self-energy according to  (see the Supporting Information for details concerning ).

While the equation for the Green function should be solved self-consistently, for the present purposes it is sufficient to replace the propagators in the self-energy with their corresponding bare ones, **g**_mol_(*z*) = (*z* – **ε**)^−1^ and . We, then, write the self-energy as  where, for instance,
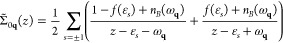
10where ε_*s*_ = ε_0_ + *sε*_1_ (ε_1_ = |**ϵ**_1_|), whereas *n*_*B*_(ω) denotes the Bose–Einstein distribution function.

In the following, our aim is to emphasize how the thermal reservoir influences the temperature dependency of the induced magnetic moment. Therefore, we investigate a molecule with an unpolarized level, that is, setting **ε**_1_ = 0. In this limit, the unperturbed Green function simplifies to **g**(*z*) = σ_0_/(*z* – ε_0_) and the electron energy ε_*s*_ → ε_0_ in the self-energy, as well as . Nevertheless, because of the form of the electron–phonon coupling it can be seen that neither Σ_0_ nor **Σ**_1_ are nonzero (see the Supporting Information). Then, for , we have
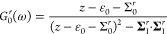
11a
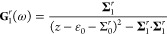
11b

First, we notice in this limit that a configuration such that *u*_0**q**_ = 0 and **u**_1**q**_ ≠ 0 may lead to a modification of the electronic state. The requirement is that . The momentum dependence of the coupling rate **u**_1**q**_ is related to the phononic polarization vector **ϵ**_**q**_ which, in turn, depends on the lattice symmetries. For instance, inversion symmetry implies that , under which conditions, then, the self-energy **Σ**_1_ = 0, hence, also **G**_1_ = 0. On this note, it is relevant to mention that chiral phonons, for which there is no inversion symmetry, would open for the possibility to generate an electronic spin-polarization, something that was considered in refs (36−38).

From the expressions in eq 11, we calculate the density of electron states , which, for *u*_0**q**_ = *u*_0_ = 0.01 and **u**_1**q**_ = 0, is plotted in Figure 2a,b as a function of the energy for temperatures corresponding to thermal energies between 1 and 20 meV. The unperturbed (bare) density of states has a single peak at the energy ε_0_ = 0.1 (red). When the electron–phonon interaction is turned on, this central peak splits into two which are located symmetrically around ε_0_. This is expected considering the poles given in eq 3. The plots in Figure 2a,b, illustrate the thermal evolution of the density of state for two different phonon velocities, (a) *c* = 0.01 and (b) *c* = 0.001. The width of the spectrum is expected to increase inversely with the velocity, since the more phonon modes contribute to the interactions with the electron the lower the velocity. This can be effectively seen in Figure 2i (dashed lines), where we have plotted the splitting between the resonances as a function of the thermal energy, illustrating that the bandwidth increases faster with lower phonon velocity.

**Figure 2:**
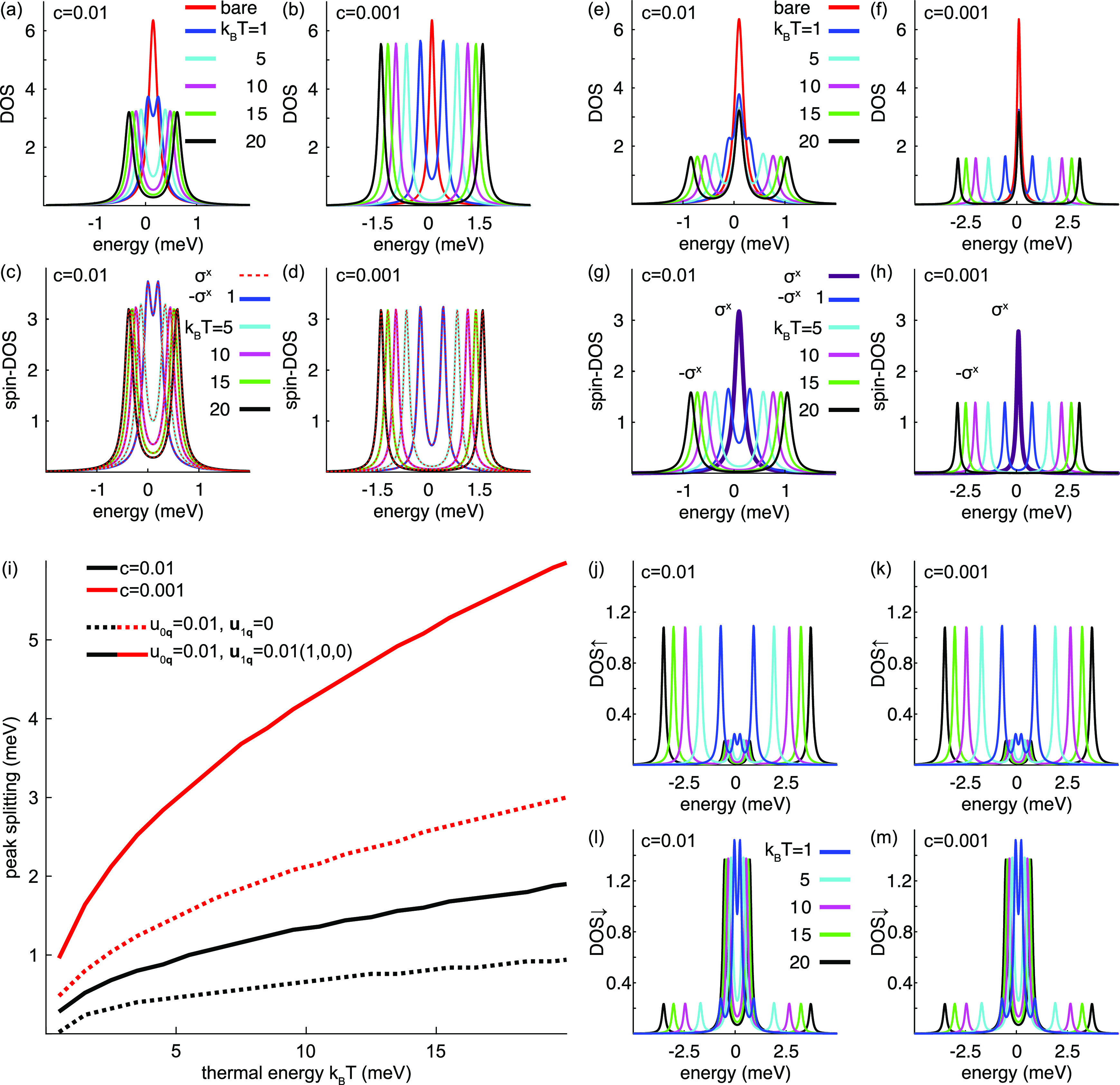
(a, b, e, f) Local densities of states (DOS) and (c, d, g, h, j–m) local spin-DOS as a function of the energy ω, for two different phonon velocities *c* = 0.01 and *c* = 0.001, and temperatures corresponding to the thermal energies *k*_*B*_*T* ∈ {1, 5, 10, 15, 20}. (i) Splitting of the density resonances in panels (a) and (b), dashed, and (e) and (f), solid, as a function of the thermal energy. Here, units are in meV. (a–d) ε_0_ = 0.1, *u*_0**q**_ = 0.01, **u**_1**q**_ = 0. The unperturbed (bare) DOS is shown for reference (red). (e–h) ε_0_ = 0.1, *u*_0**q**_ = 0.01, . The unperturbed (bare) DOS is shown for reference (red). (j, l), spin *↑*-projection, and (k, m), spin *↓*-projection, for ε_0_ = 0.1, *u*_0**q**_ = 0.01, .

Despite the splitting of the density of electron states, the spin degeneracy remains preserved. This is clear since the electron–phonon coupling only contains the spin-conserving component. This leads, trivially, to that **Σ**_1_ = 0, hence, the spin-dependent component **G**_1_ of the Green function also vanishes. In order to draw any conclusions about the spin properties under these conditions, however, we investigate the spin resolved densities of states captured in the matrix . The spin-resolved density of electron states is plotted in Figure 2c,d, illustrating the degeneracy of the spin projections.

The combination of charge and spin coupling interactions with the phonons, on the other hand, results in the emergence of two resonance peaks alongside the initial elastic peak in the density of states. This is illustrated in Figure 2e–h, where we have added the spin-dependent component . The side peaks shift to the higher energies with increasing temperature, while the central peak acquires a lowered amplitude. Also here, the lower velocity tends to induce a stronger shift of the side peaks with increasing temperature, as expected from the previous case, which is also seen in Figure 2I (solid lines).

The spin resolved densities of states are plotted in Figure 2g,h. As expected, the spin-dependent coupling  breaks the degeneracy of the electronic structure. Quite unexpectedly at first glance, on the other hand, is that the spin projections are separated into two mutually exclusive branches. Here, however, this is not surprising since the self-energies  and  are both proportional to  and , which leads to that  can be partitioned into
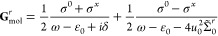
12where δ > 0 is infinitesimal.

This partitioning makes it clear that one central resonance is located at the elastic energy ω = ε_0_, whereas the other resonances are found at the condition , an equation which in the current approximation has two solutions. In Figure 2g,h, the resonances corresponding to the first and second contributions are signified by ±σ^*x*^.

The associated molecular magnetic moment  resulting from these conditions is given by
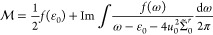
13In this result, the first contribution results from the integral , which corresponds to the first term in eq 12, whereas the second arises from the analogous calculation of the second term in eq 12. While this moment is, in general, nonvanishing, it undergoes a sign change at a finite temperature *T*_xo_. This is understood by the opposite signs of the two contributions constituting  in eq 13; recall that . Here, the first contribution, which is positive, dominates the magnetic moment at low temperature, see Figure 3. Put simply, in the main panel of Figure 3 it can be seen that whereas the central resonance at ω = ε_0_ is nearly fully occupied, the side resonances are only partially occupied. Since the former and latter resonances add positively and negatively, respectively, to the total moment, the moment is positive at sufficiently low temperature. This property is corroborated by our computations of the magnetic moment, see Figure 4a, which displays  as a function of the temperature for different phonon velocities *c*. It should also be observed that  as the electron–phonon coupling *u*_0_ → 0.

**Figure 3:**
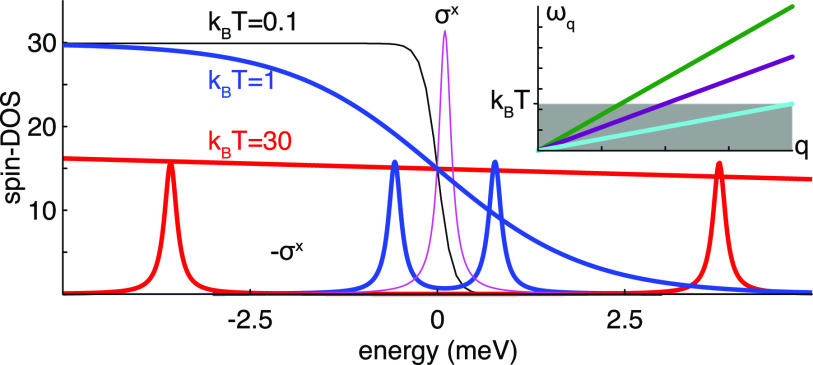
Influence of the temperature on the magnetic moment, both with respect to the shifts of the inelastic resonances and the thermal occupation factor (Fermi–Dirac distribution function). At low temperatures (blue), the inelastic resonances are strongly asymmetrically occupied while the occupation becomes symmetrized with increasing temperature (red). Very low temperatures are shown for reference (black). The spin-resolved densities are given for the conditions in Figure 2h. The inset illustrates the phonon dispersions for three different velocities, (cyan) low, (purple) moderate, and (green) high, and the expected thermally occupied states (gray area) for each dispersion relation.

**Figure 4:**
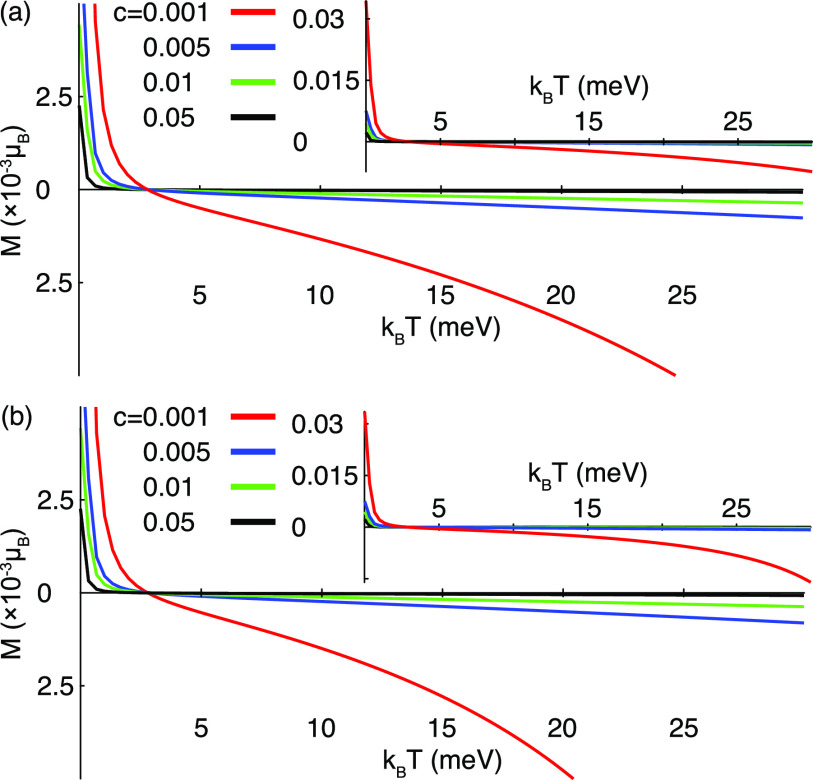
Induced magnet moment, in units of the Bohr magneton μ_*B*_, as a function of the thermal energy *k*_*B*_*T*, for the setup (a) ε_0_ = 0.1, *u*_0**q**_ = 0.01, , and (b) ε_0_ = 0.1, *u*_0**q**_ = 0.01, , and phonon velocity (blue) *c* = 0.001, (red) *c* = 0.005, (black) *c* = 0.01, and (green) *c* = 0.05 [units: meV].

With increasing temperature, the occupations of all resonances increase; however, while the occupation of the central resonance is marginally increased, the side resonances approach full occupation such that the two branches cancel out each other. Nevertheless, since the overall spin-density has a slight overweight at the side resonances, this contribution eventually becomes larger than the central resonance such that the total moment changes sign at a crossover temperature *T*_xo_ and becomes negative. The sign change and negative moment is clearly illustrated in Figure 4a, which also shows that the amplitude, both positive and negative, of the moment increases with decreasing velocity *c*.

The latter observation is understood in terms of the thermally accessible energies for a given temperature, see inset of Figure 3, illustrating the phonon dispersion relations for three velocities, (blue) low, (red) moderate, and (black) high, and the expected thermally occupied states (gray area) for each dispersion relation. For phonons with low velocity, a lower temperature is required to thermally access the energies for a larger portion of the phononic **q**-vectors in reciprocal space, compared to phonons with a higher velocity. Therefore, it is not surprising that slow phonons contribute more to the limiting magnetic moments than fast phonons.

Slowly vibrating molecules, either long polymers or composed in a crystal structure that generate acoustic vibrational modes with a low group velocity, should be suitable as a vibrational background in which defects, say some radical, may be embedded. Also, structures with optical vibrational modes would be relevant, especially ones with low group velocities. The general requirement that has to be fulfilled regardless of specific phonon structure is that inversion symmetry has to be broken for an effective vibrationally assisted spin–orbit interaction to be present.

Finally, we consider the configuration with **u**_1**q**_ = *u*_0_(1, 0, 1). For these conditions, the molecular Green function can be written
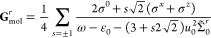
14In this setup, there is no clear separation of the central and side resonances; instead the two branches mix. In Figure 2j–m, we display plots of the spin resolved density of electron states, with *u*_*z***q**_ ≠ 0. First, one may notice that the resonances are mixtures of both spin projections. Second, it is clear that one spin branch is more heavily weighted on the side resonances, Figure 2j,k, whereas the other branch has an overweight on the central resonance, Figure 2l,m, albeit the central resonance cannot be clearly resolved.

In fact, the central resonance cannot be identified as a single resonance under the given conditions, since the electronic density comprises four distinct peaks. The four resonances can be found as the solutions to real parts of the two equations , of which the + (−) equation provides the resonances which are more heavily weighted on the side (central) resonances. In this sense, each equation corresponds to one of the two spin branches, and despite the mixing between these, one can identify a slight discrimination between them.

In this configuration, the induced molecular magnetic moment can be written , where the factor  is provided by the integral
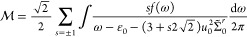
15which vanishes in the limit *u*_0_ → 0, hence emphasizing how the interactions are crucial for the formation of the magnetic moment. Again, we can identify a crossover temperature *T*_xo_ at which the total moment changes sign from positive to negative, which can be seen in Figure 4b, in which the factor  is plotted as a function of the temperature, for different phonon velocities. The mechanism for this sign change is the same as in the previous configuration. Whereas one spin-projection becomes more or less fully occupied already at low temperature and the other is only partially occupied, the latter tends to become increasingly occupied with the temperature and eventually dominates the overall magnetic moment. It can also be observed that the total magnetic moment increases when the *z*-component is added to the already existing *x*-component of the interaction parameter **u**_1**q**_. This observation is, however, trivial due to the increased number of scattering channels that are opened.

A more important, and also interesting, observation that may be made is that the temperature for the sign change of the magnetic moment appears to be universal and independent of the phonon velocity, see Figures 2j–m and 4b. This property is not surprising when considering that the sign change is a result of the competition between the two contributions, c.f., eqs13 and 15. The two contributions have equal temperature dependencies irrespective of the phonon velocity which, therefore, leads to the specific phonon distribution not impacting the temperature at which the two contributions cancel. Should the two contributions, on the other, have unequal dependencies on the phonon distribution, then the crossover temperature may vary with, e.g., the phonon velocity. Currently, we are not aware of which type of electron–phonon interactions would cause such inhomogeneous temperature dependencies; however, it is possible that structures in which the phonon modes are strongly anisotropic would open up for such properties.

In summary, we have theoretically investigated the influence of combined interactions on a localized level and demonstrated that an electronic state may become spin-polarized when coupled to reservoirs. We show that a system which is nonmagnetic whenever isolated from an surrounding environment may spin-polarize when a connection to such an environment is made. The system may spin-polarize if there are, at least, two types of interactions of which at least one has an intrinsic spin-dependence associated with it. Formally, an interaction that can be expressed as *V*_0_σ^0^ and **V**_1_·**σ** changes the electronic spectrum by . Hence, the electronic spectrum becomes spin-dependent if and only if both *V*_0_ and **V**_1_ are nonzero. Under those conditions, there is a potential for the system to acquire a nonvanishing magnetic moment.

As a corollary, we develop a theory for temperature-dependent magnetization in a molecule. We show that spin-dependent inelastic scattering, e.g., off phonons which may arise due to spin–orbit coupling,^34^ leads to breaking of the time-reversal symmetry. For this, we employ an unconventional treatment of electron scatterings off phonons by taking into account both the charge–phonon and spin–phonon couplings. While none of these couplings individually break the electronic spin degeneracy, our findings show that the combination of the two leads to a splitting of the spin channels. The effect we consider, which results in nonconserved energy collisions, originates from the interplay between spin–orbit coupling and vibrational modes. We, furthermore, demonstrate that the inelastic scattering does induce a nonzero magnetic moment of the initially unpolarized molecule, a moment for which magnitude increases with temperature but changes sign at a crossover temperature. The sign change of the magnetic moment can be explained in terms of competing influences from the relevant interactions.

Despite that we are currently aware of experimental results which comply with our theoretical discussion,^8,18,19,22,28,29^ it would intriguing to consider the effects under more extreme conditions, for instance, measurements of magnetically asymmetric thermopower or using magnetic force microscopy to measure asymmetric forces of chiral molecules attached to the surface.

## Supporting Information Available

Details of the mathematical model and derivations of the electronic Green functions and magnetization (PDF)
Transparent Peer Review report available (PDF)
