# Supplementary material for: Emergent Magnetism as a Cooperative Effect of Interactions and Reservoir
Source: J Phys Chem Lett. 2023 May 30;14(22):5119–26. doi: 10.1021/acs.jpclett.3c00526 (PMC10258847; doi:10.1021/acs.jpclett.3c00526)
Supplement: Supplementary file 1 — jz3c00526_si_001.pdf [file jz3c00526_si_001.pdf]

Supporting information:
Emergent Magnetism as a Cooperative Effect of Interactions and Reservoir

M. Shiranzaei, S. Kalhöfer, and J. Fransson
Department of Physics and Astronomy, Box 516, 75120, Uppsala University, Uppsala, Sweden
(Dated: May 16, 2023)

I. MODEL AND THEORY

A system consisting of a molecule in contact with vibrational reservoir can be modeled by,

$$\mathcal{H} = \mathcal{H}_{\rm mol} + \mathcal{H}_{\rm ph} + \mathcal{H}_{\text{e-ph}} \tag{S1}$$

in which $\mathcal{H}_{\rm mol}$ shows the molecule Hamiltonian. An electron in a molecule with a localized level can be described by,

$$\mathcal{H}_{\rm mol} = \psi^\dagger \varepsilon \psi \tag{S2}$$

where $\psi^\dagger = (c_\uparrow^\dagger \; c_\downarrow^\dagger)$ and $\varepsilon = \varepsilon_0\sigma^0 + \boldsymbol{\epsilon}_1\cdot\boldsymbol{\sigma}$. Here, ε_0 denotes the spin-independent electron energy, whereas the latter is a three component vector, $\boldsymbol{\epsilon}_1 = \varepsilon_\alpha \hat{\mathbf{e}}_\alpha$, in some normalized orthogonal basis $\{\hat{\mathbf{e}}_\alpha\}$, which accounts for, e.g, spin-orbit interactions and local spin-anisotropy. Furthermore, $\mathcal{H}_{\rm ph} = \sum_{\mathbf{q}} \omega_{\mathbf{q}} b_{\mathbf{q}}^\dagger b_{\mathbf{q}}$ represents the thermal reservoir in which $b_{\mathbf{q}}^\dagger$ ($b_{\mathbf{q}}$) creates (annihilates) a phonon at the energy $\omega_{\mathbf{q}} = c|\mathbf{q}|$. The electron and phonon are connected through hybridization with,

$$\mathcal{H}_{\rm hyb} = \sum_{\mathbf{q}} \psi^\dagger \mathbf{U}_{\mathbf{q}} \psi (b_{\mathbf{q}} + b_{\bar{\mathbf{q}}}^\dagger) \tag{S3}$$

where the coupling parameter is $\mathbf{U}_{\mathbf{q}} = u_{0\mathbf{q}}\sigma^0 + \mathbf{u}_{1\mathbf{q}}\cdot\boldsymbol{\sigma}$ whereas $\bar{\mathbf{q}} = -\mathbf{q}$. In addition to $u_{0\mathbf{q}}$ which defines a generic coupling between charge and vibrational modes, $\mathbf{u}_{1\mathbf{q}}$ denotes SOC-assisted vibrational coupling [1, 2] . Here, σ^0 and $\boldsymbol{\sigma}$ denote the 2×2 identity and vector of Pauli matrices, respectively.

A. Magnetic moment and electron Green's function

The purpose here is to evaluate the magnetic moment $\langle \mathbf{M}_{\rm mol}\rangle$ of an electron in the molecule, which can be related to the retarded Green's function $\mathbf{G}^r_{\rm mol}$ through the relation

$$\langle \mathbf{M}_{\rm mol}\rangle = -{\rm Im}\int f(\omega)\; {\rm sp}\; \boldsymbol{\sigma}\mathbf{G}^r_{\rm LS}(\omega)\frac{d\omega}{2\pi} \tag{S4}$$

where sp denotes the trace over spin $1/2$ space whereas $f(\omega)$ is the Fermi-Dirac distribution function. The retarded Green's function is calculated in terms of the Dyson-like equation as,

$$\mathbf{G}_{\rm LS} = \mathbf{g}_{\rm LS} + \mathbf{g}_{\rm LS}\boldsymbol{\Sigma}\mathbf{G}_{\rm LS}, \tag{S5}$$

where $\mathbf{g}_{\rm LS} = \mathbf{g}_{\rm LS}(z) = (z-\varepsilon)^{-1}$, $z\in\mathbb{C}$ is the bare Green's function obtained by $\mathcal{H}_{\rm mol}$, and, here, $\boldsymbol{\Sigma}$ denotes the self-energy caused by interactions between the vibrational modes and electron. Now, to evaluate the impact of vibrational modes up to the first-order, we consider the simplest Feyman diagrams for the electron scattering of phonons in self-energy [1]. By applying the equation of motion on the Green's function $\mathbf{G}_{\rm LS}(t,t') = (-i)\langle {\rm T}\psi(t)\psi^\dagger(t')\rangle$, the self-energy $\boldsymbol{\Sigma} = \sum_{\mathbf{q}} \mathbf{U}_{\mathbf{q}}\tilde{\Sigma}(z)\mathbf{U}_{\bar{\mathbf{q}}}$ is a 2×2-matrix given by the exchange loop

$$\tilde{\boldsymbol{\Sigma}}(z) = \frac{1}{\beta}\sum_\nu \mathbf{G}_{\rm LS}(z-z_\nu)\mathcal{D}(z_\nu), \tag{S6}$$

where $\beta = 1/k_BT$ defines the thermal energy in terms of the Boltzmann constant k_B and temperature T. In the second order approximation, the self-energy is evaluated by using the bare Green's functions of both electrons and phonons, here given by

$$\mathbf{g}_{\rm LS}(z) = \frac{(z-\varepsilon_0)\sigma^0 + \boldsymbol{\epsilon}_1\cdot\boldsymbol{\sigma}}{(z-\varepsilon_0)^2 - \epsilon_1^2}, \tag{S7a}$$

$$\mathcal{D}_0(\mathbf{q},z) = \frac{2\omega_{\mathbf{q}}}{z^2-\omega_{\mathbf{q}}^2}, \tag{S7b}$$

respectively, where $\epsilon_1 = |\boldsymbol{\epsilon}_1|$. Inserting Eqs. (S7a) and (S7b) into Eq. (S6) leads to

$$\tilde{\Sigma}_{\mathbf{q}}(z) = \tilde{\Sigma}_{0\mathbf{q}}\sigma^0 + \tilde{\boldsymbol{\Sigma}}_{1\mathbf{q}}\cdot\boldsymbol{\sigma}, \tag{S8}$$

in which,

$$\tilde{\Sigma}_{0\mathbf{q}}(z) = \frac{1}{2}\sum_{s=\pm1}\left(\frac{1-f(\varepsilon_s)+n_B(\omega_{\mathbf{q}})}{z-\varepsilon_s-\omega_{\mathbf{q}}} + \frac{f(\varepsilon_s)+n_B(\omega_{\mathbf{q}})}{z-\varepsilon_s+\omega_{\mathbf{q}}}\right), \tag{S9a}$$

$$\tilde{\boldsymbol{\Sigma}}_{1\mathbf{q}}(z) = \frac{\hat{\boldsymbol{\epsilon}}_1}{2}\sum_{s=\pm1} s\left(\frac{1-f(\varepsilon_s)+n_B(\omega_{\mathbf{q}})}{z-\varepsilon_s-\omega_{\mathbf{q}}} + \frac{f(\varepsilon_s)+n_B(\omega_{\mathbf{q}})}{z-\varepsilon_s+\omega_{\mathbf{q}}}\right), \tag{S9b}$$

where $\varepsilon_s = \varepsilon_0 + s\varepsilon_1$ and $\hat{\boldsymbol{\epsilon}}_1 = \boldsymbol{\epsilon}_1/\varepsilon_1$, whereas $n_B(\omega)$ denotes the Bose-Einstein distribution function.

B. Limit of vanishing spin-orbit coupling

In the limit of vanishing static spin-orbit coupling, $\boldsymbol{\epsilon}_1 = 0$, the unperturbed Green's function, Eq. (S7a) reduces to,

$$\mathbf{g}_{\rm LS}(z) = \frac{\sigma^0}{z-\varepsilon_0}, \tag{S10}$$

giving the bare density of electron states

$$n_0(\omega) = -\frac{1}{\pi}{\rm sp}\;{\rm Im}\;\mathbf{g}^r_{\rm LS}(\omega) = 2\delta(\omega-\varepsilon_0). \tag{S11}$$

In which $\mathbf{g}^r_{\rm LS}$ is obtain by $z \to \omega + i0^+$. In this limit also, the effective electron-phonon interaction is given by

$$\tilde{\Sigma}_{0\mathbf{q}} = \sum_{\mathbf{q}}\left(\frac{1-f(\varepsilon_0)+n_{\rm B}(\omega_{\mathbf{q}})}{z-\varepsilon_0-\omega_{\mathbf{q}}} + \frac{f(\varepsilon_0)+n_{\rm B}(\omega_{\mathbf{q}})}{z-\varepsilon_0+\omega_{\mathbf{q}}}\right) \tag{S12}$$

while $\tilde{\mathbf{\Sigma}}_{1\mathbf{q}} = 0$. The dressed retarded Green's function is obtained from $\mathbf{G}^r_{\rm LS} = (\mathbf{g}^{-1}_{\rm LS} - \Sigma)^{-1}$ and written in the form $\mathbf{G}^r_{\rm LS} = G^r_0\sigma^0 + \mathbf{G}^r_1\cdot\boldsymbol{\sigma}$ where

$$G^r_0(\omega) = \frac{\omega-\varepsilon_0-(u_0^2+|\mathbf{u}_1|^2)\tilde{\Sigma}_0}{[\omega-\varepsilon_0-(u_0^2+|\mathbf{u}_1|^2)\tilde{\Sigma}_0]^2-4u_0^2|\mathbf{u}_1|^2\tilde{\Sigma}_0^2}, \tag{S13a}$$

$$\mathbf{G}^r_1(\omega) = \frac{2u_0\tilde{\Sigma}_0\mathbf{u}_1\cdot\boldsymbol{\sigma}}{[\omega-\varepsilon_0-(u_0^2+|\mathbf{u}_1|^2)\tilde{\Sigma}_0]^2-4u_0^2|\mathbf{u}_1|^2\tilde{\Sigma}_0^2}, \tag{S13b}$$

where it is understood that the retarded form of $\tilde{\Sigma}_0$ is used. It is worth to notice that $G_0 = {\rm sp}\,\mathbf{G}^r_{\rm LS}/2$ and $\mathbf{G}_1 = {\rm sp}\,\boldsymbol{\sigma}\mathbf{G}^r_{\rm LS}/2$.

Here, we define the spin-resolved density of electrons states by

$$\rho(\omega) = -\frac{1}{\pi}{\rm Im}\,\mathbf{G}^r_{\rm LS}(\omega), \tag{S14}$$

which is a 2×2-matrix. The projections of the Green's function into charge (G_0) and spin ($\mathbf{G}_1$) components, furthermore, implies that the density of electron states can be expresses solely in terms of G_0, since ${\rm sp}\,\mathbf{G}_{\rm LS} = 2G_0$, giving

$$n(\omega) = -\frac{2}{\pi}\,{\rm Im}\,G^r_0(\omega). \tag{S15}$$

Likewise, the magnetic moment $\langle\mathbf{m}\rangle$ is given by the relation

$$\langle\mathbf{m}\rangle = -\frac{1}{\pi}{\rm Im}\int f(\omega)\mathbf{G}^r_1(\omega)d\omega, \tag{S16}$$

The expression of $\mathbf{G}^r_1$, Eq. (S13b), suggests that it is the combination of both non-vanishing spin-conservative coupling u_0 and and non-vanishing spin-dependent coupling $\mathbf{u}_1$ that leads to an induced magnetic moment in the system.

Further stressing importance of that both couplings are necessary in this context for the emergence of a magnetic moment is provided by the form of the self-energy. We maintain the limit $\varepsilon = \varepsilon_0\sigma^0$, such that the self-energy component $\tilde{\mathbf{\Sigma}}_1 = 0$. Nevertheless, because of the form of the electron-phonon coupling it can be seen that

$$\Sigma_0 = \sum_{\mathbf{q}}(u_{0\mathbf{q}}u_{0\bar{\mathbf{q}}} + \mathbf{u}_{1\mathbf{q}}\cdot\mathbf{u}_{1\bar{\mathbf{q}}})\tilde{\Sigma}_{0\mathbf{q}}, \tag{S17a}$$

$$\mathbf{\Sigma}_1 = \sum_{\mathbf{q}}(u_{0\mathbf{q}}\mathbf{u}_{1\bar{\mathbf{q}}} + \mathbf{u}_{1\mathbf{q}}u_{0\bar{\mathbf{q}}} + i\mathbf{u}_{1\mathbf{q}}\times\mathbf{u}_{1\bar{\mathbf{q}}})\tilde{\Sigma}_{0\mathbf{q}}. \tag{S17b}$$

Hence, for the spin component of the self-energy, $\mathbf{\Sigma}_1$, to be non-zero, it is required that not only the exchange loop $\tilde{\Sigma}_{0\mathbf{q}}$ is not identically zero. Both couplings $u_{0\mathbf{q}}$ and $\mathbf{u}_{1\mathbf{q}}$ have to be non-zero.

[1] J. Fransson, Vibrational origin of exchange splitting and chiral-induced spin selectivity, Phys. Rev. B **102**, 235416 (2020).

[2] T. K. Das, F. Tassinari, R. Naaman, and J. Fransson, Temperature-dependent chiral-induced spin selectivity effect: Experiments and theory, The Journal of Physical Chemistry C **126**, 3257 (2022).
