# Supplementary material for: Emergent Magnetism as a Cooperative Effect of Interactions and Reservoir
Source: J Phys Chem Lett. 2023 May 30;14(22):5119–26. doi: 10.1021/acs.jpclett.3c00526 (PMC10258847; doi:10.1021/acs.jpclett.3c00526)
Supplement: Supplementary file 2 — jz3c00526_si_002.pdf [file jz3c00526_si_002.pdf]

jz-2023-005265.R1

Name: Peer Review Information for "Emergent Magnetism as a Cooperative Effect of Interactions and Reservoir"

First Round of Reviewer Comments

Reviewer: 1

Comments to the Author

The authors present a theoretical study of the coupling of electrons in closed-shell molecules to a bosonic bath. They apply a Green's function approach to study the behavior of the electronic and spin density of states under the influence of the bath and find that a net magnetic moment may arise from an asymmetric thermal population of the electron-boson resonances. The authors present calculations for the example where the boson bath is given by phonons with linear dispersion on the level of a theoretical model.

In my opinion, the main result of the paper, the description of emerging magnetism from molecule-bath interactions, is a significant advance in the field of molecular magnetism and further interesting to a broad audience working on spin-phonon coupling. There are a number of questions and comments that I believe the authors should address however, before proceeding to publication.

1) Page 4: The formalism starts with a general expression for an interaction V*sigma. While I understand that the authors want to keep the formalism general, an example for what type of physical mechanism this could be would be helpful.

2) Eqs. (8), (14), (16): Over the course of the derivation of the magnetic moment, it becomes hard to follow in which parts of the expressions the different contributions of the coupling to the bath are contained. It would be good to have that pointed out at appropriate places in the manuscript, for example by taking the limit V->0 and showing which contributions lead to M->0.

3) Fig. 2: The plots are a bit cumbersome to disentangle, also because they are tiny in printout. It would be helpful to see the trend plotted directly, instead or in addition to the spectra, e.g. the splitting as a function of temperature.

4) I found it at times difficult to connect the formalism to a concrete physical picture. When discussing the electronic states coupling to phonons with linear dispersion (e.g. Fig. 3), what physical system could this be applied to? Molecules in a liquid? Defects in a solid? Electronic states in a large molecule coupling its own vibrational bath? I think it would help a lot to point out one or two examples.

5) Connected to the above point: In Fig. 3, phonons with linear dispersion are assumed. How would the results change, if optical phonons are considered, which generally have very small group velocities.

6) Page 8: The authors note that Eq. (9) contains a vibrationally induced spin-orbit coupling. This sounds very familiar to a recent study [Maslov, Phys. Rev. B 101, 184104 (2020)], which describes spin-orbit coupling arising from the effect of a phononic bath. It would be appropriate if the authors clarified to what extent these mechanisms are related/differ from each other.

7) Page 10: The authors note that chiral phonons can possibly generate an electronic spin polarization. This has indeed been studied very recently in solids and might be appropriate to refer to in this context. See, e.g., [Juraschek, Phys. Rev. Research 4, 013129 (2022)] and [Geilhufe, Phys. Rev. B 107, L020406 (2023)].

Minor points:

i) Fig. 1: The inset equations contain a and a^dagger for the phonons instead of b and b^dagger, as in the main text.

ii) Fig. 4: What unit is the magnetic moment given in?

Reviewer: 2

Comments to the Author

This is a provocative article in the sense that the authors claim that a confluence of internal motion and coupling to a bath can induce an emergent magnetic moment and break time-reversibility. In a sense, we know that something like this must be true from the Ising model of magnetism, but here the claim is that such physics can occur with only a small finite system coupled to a noninteracting bath.

I'd like to say that I understood all of this article, but I fear that the authors have strongly overestimated their audience.

For the first part of the article, I think I follow but I am left somewhat unsatisfied. The authors never discuss how Sigma is constructed and what form Sigma can take that is still compatible with time-reversibility: is this not important? The authors do not address what happens if I consider applying the time-reversal operator to the solution? This would greatly help me understand their symmetry breaking result (i.e. why a magnetic moment appears).

For the second part of paper, I must admit that I didn't follow nearly as much. Likely because of the space limitations, the details are really packed into this paper. I feel the paper would be far better if the authors were to put almost all of the math in a long supplementary material file (a la PRL), where they slowly define the Hamiltonian, go over their choice of \Sigma, introduce subsections and headings to make clear what is the focus of each equation, and then slowly describe their theoretical and graphical results one at a time (not with 6 tiny figures in one row).

The thing is that all of this work is based on equilibrium greens functions, so everything should be accessible to a general audience who do now know about NEGFs. The authors need to provide more intuition for the reader to appreciate the results here.

Author's Response to Peer Review Comments:

Dear Prof. Editor,

We herewith resubmit our manuscript *Emergent Magnetism as a Cooperative Effect of Interactions and Reservoir* by M. Shiranzaei, S. Kalhöfer, and J. Fransson, for consideration for publication in the Journal of Physical Chemistry Letters.

It is a pleasure to see that both reviewers have an essentially positive attitude towards the manuscript, although there are few issues they ask us to consider, none of which adverse to the content of the manuscript. We provide a detailed list of our responses below.

Based on these arguments, we are firmly convinced that the paper is suitable for publication in the Journal of Physical Chemistry Letters.

Yours sincerely,

Mahroo Shiranzaei, Sebastian Kalhöfer, and Jonas Fransson

Response to Reviewer 1

We are pleased to see that the reviewer is generally positive to our manuscript and that the results we wish to communicate are well recognized.

1. Reviewer: *Page 4: The formalism starts with a general expression for an interaction V*sigma. While I understand that the authors want to keep the formalism general, an example for what type of physical mechanism this could be would be helpful.*

   Our response: We appreciate the reviewer's request and we understand that we have been somewhat short in that respect. Typical mechanisms that we have in mind here are Coulomb and electron-phonon interactions, but may also arise from light-matter interactions. We have added some detail about the mechanisms we have in mind in this context.

2. Reviewer: *Eqs. (8), (14), (16): Over the course of the derivation of the magnetic moment, it becomes hard to follow in which parts of the expressions the different contributions of the coupling to the bath are contained. It would be good to have that pointed out at appropriate places in the manuscript, for example by taking the limit $V \to 0$ and showing which contributions lead to $M \to 0$.*

   Our response: We appreciate this comment and recognize that some more detail will enhance the readability of the paper. We have worked through the text in this derivation and improved on the justifications and reasons for obtaining certain results.

   We noticed some typos around Eq. (8), which would have lead to make it difficult to follow the derivation. The corrected derivation should be more straight forward to follow and, in particular, our conclusions remain unchanged.

   Following the suggestion made by the reviewer we have also added comments, where appropriate, concerning the necessity that the interactions $\mathbf{V}$, or $\boldsymbol{\Sigma}$, are non-zero in order for the magnetic moment to arise.

3. Reviewer: *Fig. 2: The plots are a bit cumbersome to disentangle, also because they are tiny in printout. It would be helpful to see the trend plotted directly, instead or in addition to the spectra, e.g. the splitting as a function of temperature.*

   Our response: The reviewer is correct in that the plots in Fig. 2 can be improved. For this reason, we have worked with the layout of the panels, as well as, added a fourth panel showing the splitting of the resonances as function of the temperature for the cases plotted in Fig. 2 (a), (b), (e), and (f). We hope that this panel will assist the reader to comprehend the presented data in a better way.

4. Reviewer: *I found it at times difficult to connect the formalism to a concrete physical picture. When discussing the electronic states coupling to phonons*

with linear dispersion (e.g. Fig. 3), what physical system could this be applied to? Molecules in a liquid? Defects in a solid? Electronic states in a large molecule coupling its own vibrational bath? I think it would help a lot to point out one or two examples.

Our response:

The reviewer correctly points out that there is missing pieces about concrete configurations where our predictions may be observed. A necessary condition for the vibrationally induced spin-orbit coupling is that the inversion symmetry is broken. However, whenever this condition is satisfied, one should, in principle, be able to observe emergent magnetic states for defects in solids, molecules in liquids, and even in structures withholding their own vibrational modes and coupled to an environment. An example of the latter is provided by chiral molecules adsorbed on metals, where the molecular vibrations and coupling to the metal cooperatively generate a magnetic state in the molecule, see, e.g., *Nat. Comms.*, **2017** *8*, 14567 and Nano Lett. **2021**, *21*, 3026. We have, thanks to the reviewer, added a paragraph where we discuss possible concrete examples where our prediction might be observed.

5. Reviewer: *Connected to the above point: In Fig. 3, phonons with linear dispersion are assumed. How would the results change, if optical phonons are considered, which generally have very small group velocities.*

   Our response: We thank the reviewer for raising this issue, since it is a fair question and may be relevant in our discussion. It can be seen, nonetheless, from the abstract discussion in the first part of the manuscript that our results qualitatively do not depend on the specific nature of the Bosonic energy spectrum and so forth. There would be quantitative changes, but we can notice in our simulated results that the lower the group velocity of the phonons, the larger the impact on the induced magnetic moment. It would, by this logic, be expected that optical phonon may provide an efficient source for magnetization. We have added some comments about this conjecture in the discussion.

6. Reviewer: *Page 8: The authors note that Eq. (9) contains a vibrationally induced spin-orbit coupling. This sounds very familiar to a recent study [Maslov, Phys. Rev. B 101, 184104 (2020)], which describes spin-orbit coupling arising from the effect of a phononic bath. It would be appropriate if the authors clarified to what extent these mechanisms are related/differ from each other.*

   Our response: We thank the reviewer for pointing out this article, it is one that we have missed. It supports the concept of a vibrationally induced spin-orbit coupling, just like the results in Refs. [33,34] of the previous version of the manuscript, which is something that we exploit in our study. The results are all based on a broken inversion symmetry to be viable and are in

this sense quite analogous to one another. We have added this reference and mention it along with Refs. [33,34].

7. Reviewer: *Page 10: The authors note that chiral phonons can possibly generate an electronic spin polarization. This has indeed been studied very recently in solids and might be appropriate to refer to in this context. See, e.g., [Juraschek, Phys. Rev. Research 4, 013129 (2022)] and [Geilhufe, Phys. Rev. B 107, L020406 (2023)].*

   Our response: We thank the reviewer for correctly pointing out these references which are relevant to also mention, and which is also done in the updated version of the manuscript.

8. Reviewer: *Minor points: i) Fig. 1: The inset equations contain a and $a^\dagger$ for the phonons instead of b and $b^\dagger$, as in the main text. ii) Fig. 4: What unit is the magnetic moment given in?*

   Our response: i) We thank the reviewer pointing out this typo, which is now corrected. ii) Concerning the magnetic moment, we have simply plotted the values of the integral in Eq. (14), which is unit less. However, this should be scaled with $g\mu_B/\hbar$, hence, suggesting that the induced moment is about an order of magnitude larger than the nuclear moment. We have included the scaling unit in the figure.

Response to Reviewer 2

We appreciate that the reviewer feels provoked by the content of this manuscript. While our intention is not to provoke *per se*, we are also stunned by the possibilities that are opened by our results. We are, moreover, pleased that the reviewer is generally positive to our manuscript provided that we work to improve the presentation. However, as we are optimistic we believe that the audience is more fit to comprehend our results than what might be anticipated. It is, nevertheless, important to display the crucial results when relevant.

1. Reviewer: *The authors never discuss how Sigma is constructed and what form Sigma can take that is still compatible with time-reversibility: is this not important? The authors do not address what happens if I consider applying the time-reversal operator to the solution? This would greatly help me understand their symmetry breaking result (i.e. why a magnetic moment appears).*

   Our response: In the first part of the manuscript, we intentionally do not specify the origin of the self-energy $\boldsymbol{\Sigma}$ except the fact that it can be written in terms of the charge and spin components as $\boldsymbol{\Sigma} = \Sigma_0\sigma^0 + \boldsymbol{\Sigma}_1 \cdot \boldsymbol{\sigma}$. Such a decomposition can always be made for a 2×2-matrix. The reason for not being specific is that we try to be as general as we can and not make assumptions about the nature of the interactions. In the second part of the

manuscript, on the other hand, we have specified the origin of the self-energy as the electron-phonon exchange loop which can be found in nearly any text book on many-body physics.

Concerning the time-reversal symmetry, it is actually quite straight forward to see that the Green function of the type given in Eq. (11) of the manuscript preserves time-reversal symmetry only under certain conditions. First, this Green function $\mathbf{G}^r_{\text{mol}}$ is written as $\mathbf{G}^r_{\text{mol}} = G^r_0\sigma^0 + \mathbf{G}^r_1 \cdot \boldsymbol{\sigma}$. Furthermore, since $\mathbf{G}^r_1 = \text{sp}\boldsymbol{\sigma}\mathbf{G}^r_{\text{mol}}/2$ defines the spin-component of the Green function, it is, by definition, odd in time. Hence, the Green function is time-reversal symmetric whenever the self-energy component $\boldsymbol{\Sigma}_1 = 0$, otherwise it is not. We discuss the consequences of $\boldsymbol{\Sigma}_1 \neq 0$ since this is a necessary condition for the environment to stabilize the magnetic moment, c.f., $\langle\mathbf{M}\rangle \sim -\text{Im}\int f(\omega)\mathbf{G}^r_1(\omega)d\omega$, and provide an example for such a possibility in terms of the spin-dependent electron-phonon coupling.

Time-reversal symmetry is certainly important. However, one has to appreciate that the coupling to the Bosonic reservoir and introduction of inelastic scattering already breaks the time-reversal symmetry since this is a source for damping. During the damping process the electronic subsystem may acquire a magnetic state, and this is what emerges in the solution. We understand that we have been far too abstract in the presentation of this material, that we have not provided obvious connections to real physical and chemical systems. This is something we have worked to improve in the updated version of the manuscript, pointing out the sources of interactions (Coulomb, electron-phonon, and light-matter interactions) that may be comprised in $\boldsymbol{\Sigma}$. We have also emphasized in the text the necessity that $\boldsymbol{\Sigma}_1$ is non-zero for the emergence of a non-vanishing magnetic moment.

It can be noticed from the discussion in *Phys. Rev. B*, **2020**, *102*, 235416 – Ref. [33] in the previous version of the manuscript – that while the spin-dependent electron-phonon coupling in the Hamiltonian preserves time-reversal symmetry, it is by the inclusion of the inelastic scattering originating from the phonons, that the time-reversal symmetry breaks.

2. Reviewer: *For the second part of paper, I must admit that I didn't follow nearly as much. Likely because of the space limitations, the details are really packed into this paper. I feel the paper would be far better if the authors were to put almost all of the math in a long supplementary material file (a la PRL), where they slowly define the Hamiltonian, go over their choice of* Σ*, introduce subsections and headings to make clear what is the focus of each equation, and then slowly describe their theoretical and graphical results one at a time (not with 6 tiny figures in one row).*

   Our response: The reviewer is correct in that the second part of the paper requires quite a bit of the reader and we have, therefore upon the reviewer's suggestion, moved parts of the derivation to a Supplement for the benefit of readability but also allowing for a more verbose presentation or our results.

3. Reviewer: *The thing is that all of this work is based on equilibrium greens functions, so everything should be accessible to a general audience who do now know about NEGFs. The authors need to provide more intuition for the reader to appreciate the results here.*

   Our response: Yes, this is surely a fair observation and comment by the reviewer. Nevertheless, since the lesser (greater) Green functions are more generally applicable than is the retarded (advanced), one could certainly use this formalism also in this situation. However, since we can write everything we need in terms of the retarded Green function, given the equilibrium conditions, we have followed the reviewer's request and removed all reference to the lesser (greater) Green function.

Summary of changes

Page 4: Text added:

We notice that, the nature of the interactions may comprise electron-electron and electron-phonon type of interactions, but may also stem from, e.g., light-matter interactions of different kinds. The important feature that has to be recognized in this context is the presence of components with different symmetries with respect to the spin degrees of freedom. In this sense we stipulate that there should be one component that couples to the electron charge and one to the electron spin.

Page 4: Text added:

$\langle \mathbf{m} \rangle = -\mathrm{Im} \int f(\omega) \mathrm{sp} \boldsymbol{\sigma} \mathbf{G}^r_{\mathrm{LS}}(\omega) d\omega/2\pi$, where $\mathbf{G}^r_{\mathrm{LS}}$ denotes the retarded Green function and $f(\omega)$ is the Fermi-Dirac distribution function

Page 6: Text added:

It should be noticed that the vibrationally induced spin-orbit coupling $\mathbf{u}_{1\mathbf{q}}$, in general, is non-vanishing in structures with broken inversion symmetry. Hence, in the present discussion we assume that those requirements are met in the structure.

Page 8: Text added:

$\Gamma_s \rightarrow \gamma$. Implementing the observation, if can be seen that ...

Page 8: Text added:

, where the self-energy $\boldsymbol{\Sigma}$ accounts for the phonon assisted absorption and emission processes illustrated in Fig. 1. The structure of the model allows us to factorize the self-energy according to $\boldsymbol{\Sigma} = \sum_{\mathbf{q}} \mathbf{U}_{\mathbf{q}} \tilde{\boldsymbol{\Sigma}} \mathbf{U}_{\bar{\mathbf{q}}}$ (see the Supporting information for details concerning $\tilde{\boldsymbol{\Sigma}}_{\mathbf{q}}$).

Page 9: Text added:

neither Σ_0 nor $\boldsymbol{\Sigma}_1$ are non-zero (see the Supporting information)

Page 9: Text added:

$\langle n_{\mathrm{mol}} \rangle = -\mathrm{Im} \int \mathrm{sp}\mathbf{G}^{r}_{\mathrm{mol}}(\omega) d\omega/2\pi = -\mathrm{Im} \int G^{r}_{0}(\omega) d\omega/\pi$

Page 10: Text added:

This can be effectively seen in Fig. 2 (i) (dashed lines), where we have plotted the splitting between the resonances as function of the thermal energy, illustrating that the band width increases faster with lower phonon velocity.

Page 10: Text added:

, which is also seen in Fig. 2 (i) (solid lines).

Page 11: Text added:

In this result, the first contribution results from the integral $-\mathrm{Im} \int f(\omega)\mathrm{sp}\boldsymbol{\sigma}(\sigma^0 + \sigma^x)(\omega - \varepsilon_0 + i\delta)^{-1} d\omega/4\pi = \int f(\omega)\delta(\omega - \varepsilon_0) d\omega/2\pi = f(\varepsilon_0)/2$, which corresponds to the first term in Eq. (12), whereas the second is arises from the analogous calculation of the second term in Eq. (12).

11: Text added:

It should also be observed that $\mathcal{M} \to 0$ as the electron-phonon coupling $u_0 \to 0$.

Page 12: Text added:

Slowly vibrating molecules, either long polymers or composed in a crystal structure that generate acoustic vibrational modes with a low group velocity should be suitable as a vibrational background in which defects, say some radical, may be embedded. Also, structures with optical vibrational modes would be relevant, especially ones with low group velocities. The general requirement that has to be fulfilled regardless of specific phonon structure is that inversion symmetry has to be broken for an effective vibrationally assisted spin-orbit interaction to be present.

13: Text added:

which vanishes in the limit $u_0 \to 0$, hence emphasizing how the interactions are crucial for the formation of the magnetic moment.

Figure 2: has been reconfigured and a new panel has been added, and corresponding changes in the figure caption. Also, the text "(i) Splitting of the density resonances in panels (a), (b), (e), and (f), as function of the thermal energy. Here, units are in meV." is added.

Figure 4: has been updated with units and in figure caption the text ", in units of the Bohr magneton μ_B," is added.

- A Supporting Information file is added to the submission.

jz-2023-005265.R2

Name: Peer Review Information for "Emergent Magnetism as a Cooperative Effect of Interactions and Reservoir"

Second Round of Reviewer Comments

Reviewer: 1

Comments to the Author

The authors have made changes to their manuscript that improves the understanding of the physical mechanism. The supplementary information brings more insight into the details of the formalism and is very much appreciated.

I can now recommend the manuscript for publication in its current state.

Just one last thing I noticed: in Fig. 2(i), there are four graphs: red, red dashed, black, and black dashed, but the legends show red, black, magenta, and magenta dashed. I guess that's a mistake, but one easy to correct.

Author's Response to Peer Review Comments:

Dear Professor Editor,

We are delighted to see that our manuscript is about to become accepted. We have made changes in the manuscript, figure, and supporting information as requested by the reviewer and you. We believe that the material is now set for publication.

Yours sincerely,

Jonas Fransson.
